# A Study on Microstructure, Residual Stresses and Stress Corrosion Cracking of Repair Welding on 304 Stainless Steel: Part II-Effects of Reinforcement Height

**DOI:** 10.3390/ma13112434

**Published:** 2020-05-26

**Authors:** Xiaodong Hu, Hao-Yong Jiang, Yun Luo, Qiang Jin, Wei Peng, Chun-Mei Yi

**Affiliations:** 1 College of Mechanical and Electronic Engineering, Shandong University of Science and Technology, Qingdao 266590, China; huxdd@163.com (X.H.); 17854252995@163.com (H.-Y.J.); 2 Shandong Yuhuang Chemical (Group) Co., Ltd., Heze 274032, China; 3 College of New Energy, China University of Petroleum (East China), Qingdao 266555, China; jinqiangupc@163.com (Q.J.); pengwei1124@163.com (W.P.); 4 School of Petroleum Engineering, China University of Petroleum (East China), Qingdao 266555, China; 5 Shandong Meiling Chemical Equipment Co., Ltd., Zibo 255430, China; sdmljsk@163.com

**Keywords:** 304 stainless steel, repair welding, reinforcement height, residual stresses, stress corrosion cracking

## Abstract

The repair reinforcement height is an important parameter of repair welding, which may have a great influence on structural integrity. In this paper, the effects of repair welding reinforcement height on the microstructure, microhardness, residual stresses and stress corrosion cracking (SCC) behavior of a 304 stainless steel-repaired joint were investigated by experimentation and simulation. With an increase of the repair weld reinforcement height, the *δ* ferrite content in weld and fusion zone is obviously reduced, and the ferrite shape is gradually changed from the skeleton to the worm shape. With the increase of repair welding reinforcement height, the microhardness and residual stresses decrease gradually. The tensile strength and elongation for higher repair weld reinforcement height are larger than those with lower repair weld reinforcement height. The higher the repair weld reinforcement height, the harder it is for SCC to occur. The repair welding in 304 stainless steel is recommended to be repaired no more than two times.

## 1. Introduction

In the manufacturing, installation and servicing processes of pressure vessels, some defects (cracks, corrosion pits, wear, etc.) are inevitably generated and have a great influence on the safe service of the equipment [1]. In order to prolong the service life of pressure vessel and reduce the production costs, it is necessary to repair the pressure vessel locally by repair welding in order to ensure the structural integrity of pressure vessels [2]. However, due to uneven temperature field distribution during welding, the residual stresses are generated inevitably after repair welding, which has a great influence on the later service life of the repair equipment [3]. Especially for the repaired equipment containing corrosion medium, it is prone to occur stress corrosion failure [4]. Therefore, it is very important to study the influences of different repair welding parameters on residual stress and stress corrosion sensitivity, which are of great significance to optimizing the repair welding process and improving the service life of the repair structures.

The process of repair welding is accompanied by the local heating and microstructural changes in welded joint. If the repair welding technology is improper, the local repair area may fail again in a short period of time, resulting in the scrapping of the equipment in advance [5]. It is necessary to clarify the effects of local repair welding technology on the structure integrity of repaired joint, so that the repaired joint has a better service performance. Zeinoddini et al. [6] studied the effect of repair welding on the residual stress in the welding joint area of a submarine pipeline. They found that the local repair welding has a greater effect on the tensile transverse residual stress at the original weld than the full repair welding. Increasing the number of repair welds in the same area will increase the residual stress in the repair welding. Tanaka et al. [7] also studied the effects of repair welding times on metallographic structure, grain orientation, impact properties and pitting corrosion of local repair welding zone of stainless steel. They found the higher the number of repair welds, the shorter and lesser the length and quantity of banded ferrite. Aghaali et al. [8] studied the effects of the number of repair welds on the mechanical properties of the local repair welding zone of 316L stainless steel, and found that the microhardness and impact toughness of the heat affected zone (HAZ) of the repair weld were reduced with the increase of the number of repair welds. Jiang et al. studied the effects of the repair welding width [9,10], length [11] and iterations [12] on the repaired residual stress. The residual stress of repair welding can be reduced by increasing the repair welding width and times. The effect of the repair welding length on the transverse residual stresses is larger than that on the longitudinal residual stresses. In addition, Jiang et al. [12] also investigated the effects of repair welding times on microhardness and the metallographic structure of repaired joint. With increasing repair welding times, the more spot ferrite content, the lower the residual stress and microhardness are. Dong et al. [13,14] found that the effect of repair welding on the residual stress in the local repair zone is greater than that of initial welding, and the smaller the repair welding length is, the greater the transverse residual stress is. Soanes et al. [15] optimized the repair welding process of a steam pipe from the point of view of reducing the residual stress by simulation. In summary, the repair welding technology and structure parameter have a great influence on structure integrity, via microstructure, microhardness and residual stresses, and then affect the service behavior of the repaired structure.

Stress corrosion cracking (SCC) is a main failure mode of a repaired structure in a corrosion environment. Different repair welding technologies must generate different effects on SCC behavior because of different residual stresses and microstructure distributions. Nam et al. [16] studied the effect of weld bead number on stress corrosion sensitivity of STS304 steel pipe. The stress corrosion penetration crack appeared near the weld, and the penetration crack’s generation time decreased with increasing weld bead number. With increasing repair welding times, the depth of pitting pit in repair welding zone deepens [7] and the pitting tendency of the repaired HAZ increases [8]. Xu et al. [17] studied the stress corrosion of welded joint zone of pipeline steel in H_2_S medium. The stress corrosion sensitivities of weld and HAZ are higher than those of base metal. Cleiton et al. [18] found that with the increase of line energy, the *δ*-ferrite content in welding joint increases, which leads to the decrease of corrosion rate of welded joint. Chehuan et al. [19] found that secondary austenite γ^2^ has little effect on corrosion resistance of a UNS32750 duplex stainless steel welded joint. Consequently, the microstructure has a great influence on the corrosion resistance of repaired joint.

The mechanical properties, SCC behavior and service life of the repaired joint are influenced by the repair welding technology. Through a careful investigation of residual stress and SCC behavior on as-weld joints [20,21,22,23], we found that there are few studies on the residual stresses and SCC behavior via repair welding a joint experimentally. 304 stainless steel is a very popular engineering repair material due to the merits of corrosion resistance [24,25,26]. The aim of this paper is to study the effects of repair welding reinforcement height on microstructure, residual stresses and SCC behavior of a joint repair welded with 304 stainless steel. For the welding residual stress, it is very hard to clarify the residual stress distribution by experimentation, and finite element modeling (FEM) is a common method for simulating the entire residual stresses distribution of many welded structures [27,28,29,30,31,32,33,34,35,36,37]. As a sequel to Part I in which the effect of heat input was investigated, Part II focuses upon the effect of reinforcement height. An optimized repair weld reinforcement height was proposed in this study.

## 2. Experiment

### 2.1. Specimen Preparation

**Figure 1:**
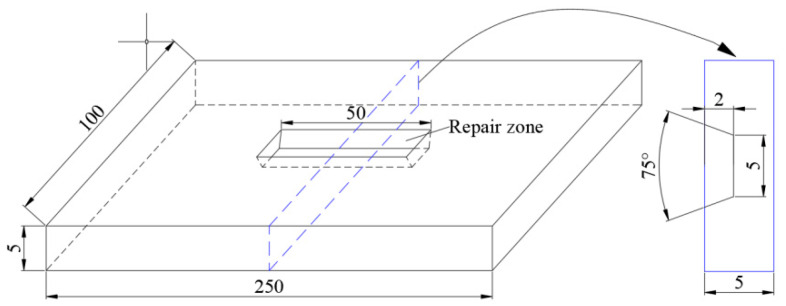
The schematic diagram of repaired specimen.

In this paper, 304 stainless steel plate with thickness of 5 mm is selected as the base metal of repair welding, and A102 electrode with diameter of 4 mm is selected as welding material because it has excellent welding performance and anti-porosity performance. The main chemical compositions of 304 stainless steel and A102 electrode are listed in Table 1. The yield strength and tensile strength are 266 and 785 MPa, respectively. The dimension of parent specimen is 250 × 100 × 5 mm. In order to make the surface defect, the groove defect is extracted by machining in the center of the sample. Figure 1 shows the schematic diagram of the repaired specimen. The defect depth is 2 mm, and the groove angle is 75°. The width defect bottom is 5 mm. The repair welding length is 40 mm.

Manual arc welding technology is used to repair the defects of the sample. The welding parameters of repaired specimens with different repair heights are listed in Table 2. The repair height (*k*) was set as 0.5, 1.0, 2.5 or 3.5 mm, respectively. For repair heights 0.5 and 1.0 mm, only a single welding pass was performed, and two welding layers were performed for other repair heights.

Before repair welding, the defects were cleaned to prevent impurities from entering the repair layer, and then we preheated the areas around defects at 200 °C to keep the base metal dry during welding. In the process of repair welding, in order to prevent excessive deformation of the specimen, fixtures should be used to fix both ends of the sample. After repair welding, the sample was cooled naturally in air.

### 2.2. Microstructure and Microhardness

The metallographic test block was cut from repaired specimen, and the cross-section was polished with sand paper from 100# to 800#. Afterwards, all metallographic samples were polished on the metallographic polisher until there were no obvious scratches on the surface of the samples. Then, the cross-section was soaked in aqua regia (HCl:HNO_3_ = 3:1) for about one minute, and then cleaned by clean water and anhydrous alcohol for morphological observation under a microscope. The microstructure of weld, fusion line and heat affected zone of metallographic specimen was observed by optical microscope, CARL ZEISS.

After observing the metallographic structure, the microhardness of the weld, fusion line and HAZ of each sample was measured by Vickers hardness tester. The load of microhardness tests was 0.02 kgf and duration time was 20 s. The hardness of each region was measured three times, and the average hardness of each region was obtained.

### 2.3. Residual Stresses Measurement

**Figure 2:**
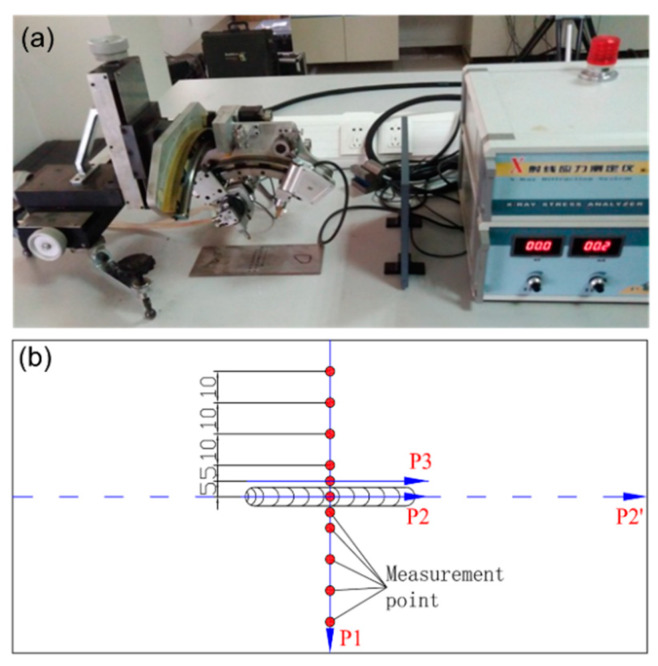
The XRD setup (**a**) and measurement paths and points (**b**).

The residual stresses were measured by X-ray diffraction (XRD) technique. The XRD measurement is based on Bragg’s law [38]. The experimental setup is shown in Figure 2a. The X-ray measurement was done by the X-350A type X-Ray stress gauge, Proto company: 11 points along P1 with an interval of 5 mm in weld and 10 mm in other area were measured, as presented in Figure 2b. Here, the path P1 is located in the center line of the upper surface perpendicular to the weld. P2 is located in the center line of the weld upper surface parallel to the weld. P3 is located in the upper surface of HAZ parallel to the weld.

### 2.4. Slow Strain Rate Tests (SSRT)

**Figure 3:**
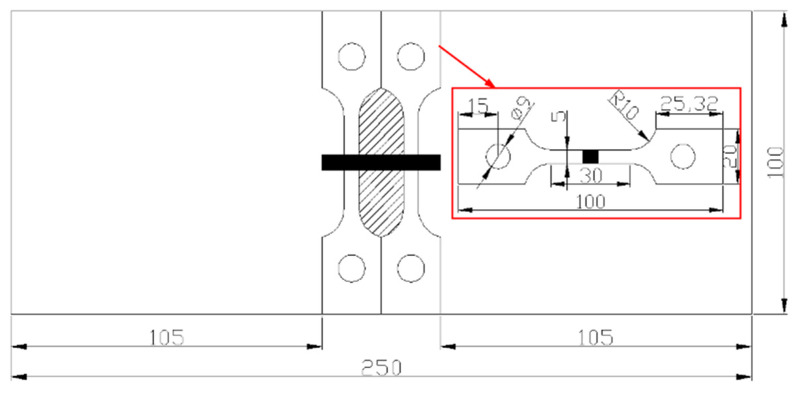
The processing position and geometry of slow strain rate test (SSRT) specimen.

After the measurement of welding residual stress, the SSRT specimens are processed. The processing position and geometry are shown in Figure 3. The weld is located at the center of the SSRT specimen, and the welding direction of repair weld is perpendicular to the tensile direction of SSRT specimen. Only specimen B1 and B4 were used to perform SSRT. Two SSRT samples were machined from the same repair welding sample, one for the tensile test in the corrosive medium and the other for the tensile test in air. In engineering practice, the reinforcement height of repair weld is usually removed to eliminate stress concentration. Therefore, the reinforcement height of the SSRT sample should be polished to be the same as the base metal.

The corrosion solution is the 3.5% NaCl solution (wt. %). The strain rate is 10^−6^ s^−1^. The test procedure contains four steps: Firstly, grind the SSRT sample with sandpaper to remove the residual knife mark in the process; then rinse with distilled water and dry it, and put it into the drier for use. Second, the SSRT sample is fixed in the solution pool; then the solution pool containing the sample is installed on the testing machine; and finally the 3.5% NaCl solution is poured into the solution pool. Third, the 300 N load is applied in advance to eliminate the clearance, and then the test begins until fracture. Finally, the fractured SSRT specimen was cleaned and blown dry. The standard distance after fracture was measured.

Stress corrosion cracking (SCC) sensitivity index *I_δ_* is calculated by:

(1) $$I_{\delta }=(1-\frac{\delta_{cor}}{\delta_{air}})\times 100\%$$

where *δ*_cor_ and *δ*_air_ are the characteristic parameters (here, it is elongation) in inert medium and air, respectively. A bigger SCC sensitivity index represents the specimen more easily having SCC. After fracture testing, the fracture surface of the specimen is cleaned by ultrasonic waves to ensure no impurity contamination. The fracture morphology was observed by scanning electron microscope.

## 3. Simulation on Residual Stresses

### 3.1. Finite Element Model

**Figure 4:**
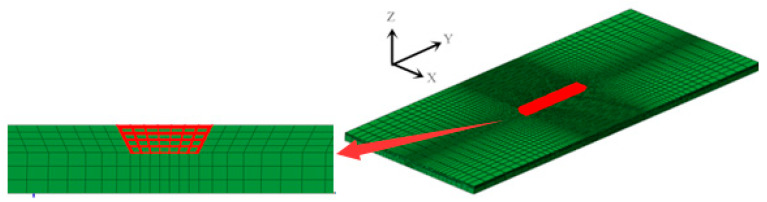
Finite element meshing of repaired specimen.

A three dimensional (3D) model was built according to the dimensions shown in Figure 1. The typical finite element meshing of whole repair specimen was shown in Figure 4. In order to reduce computational time, the meshing is dense in the weld, and then it becomes coarse, gradually, far away. The mesh independence on the calculation result has been checked. The element types for the thermal analysis and mechanical analysis are DC3D8 and C3D8R, respectively. A sequential coupling analysis combining thermal and stress analysis was used for the repair welding simulation analysis. The welding temperature analysis is conducted first, and then followed by stress analysis which employs the temperature obtained from thermal analysis.

### 3.2. Welding Temperature Analysis

In welding temperature analysis, the element birth and death technique were adopted to simulate the weld bead deposition. The welding temperature field is primarily simulated by applying a heat source for the double ellipsoidal distribution proposed by Goldak [39], which is described in Part I in detail. The moving process of double ellipsoidal heat source is realized by a user subroutine DFLUX. The temperature-dependent thermal physical properties of 304 stainless steel were listed in Part I. In Part I, the temperature field of weld section by thermal simulation was compared with weld pool morphology by experiment. The simulated weld pool profile agrees the actual morphology very well, indicating that the simulation method of temperature field in repair welding is feasible in this study.

### 3.3. Residual Stress Analysis

The repair welding residual stress is calculated by using the temperature field distribution obtained from welding temperature analysis as input data. The solid-state phase transformation was assumed to not occur during welding for 304 stainless steel. The total strain contains elastic strain, plastic strain and thermal strain, and their detailed the calculation principles were also presented in Part I. The annealing effect is considered here. The temperature-dependent mechanical properties of 304 stainless steel were listed in Part I. During the stress analysis, four end nodes on bottom surface are constrained to avoid the rigid body motion.

## 4. Results and Discussion

### 4.1. Macrostructure and Microstructure

**Figure 5:**
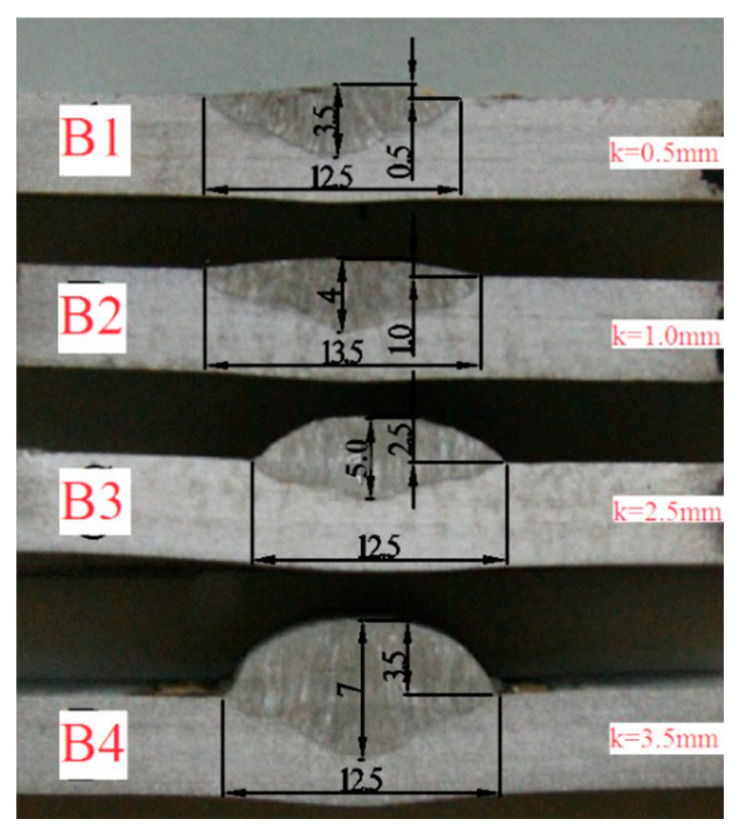
Morphology and size of the molten pool for different repair weld reinforcement heights.

The morphology and size of the molten pool for different repair weld reinforcement heights are shown in Figure 5. The welding speed for repair height 1.0 mm is smaller than that for repair height 0.5 mm. It was found that the fusion depth-widths of samples B1, B2, B3 and B4 were 3–12.5, 3–13.5, 2.5–12.5 and 3.5–12.5 mm, respectively. Sample B1 and B2 are single layer welding, and the heat input of sample B2 is larger than that of B1, resulting in samples B2 and B1 having the same penetration depth, while the melting width of sample B2 is slightly larger than that of B1. Both the sample B3 and B4 are double layer welding, and the heat input of sample B4 is larger than that of B3. Thus, the samples B4 and B3 have the same melting width, and the penetration depth of sample B4 is slightly larger than that of B3. It indicates that the penetration width and fusion width increase with the increase of heat input.

**Figure 6:**
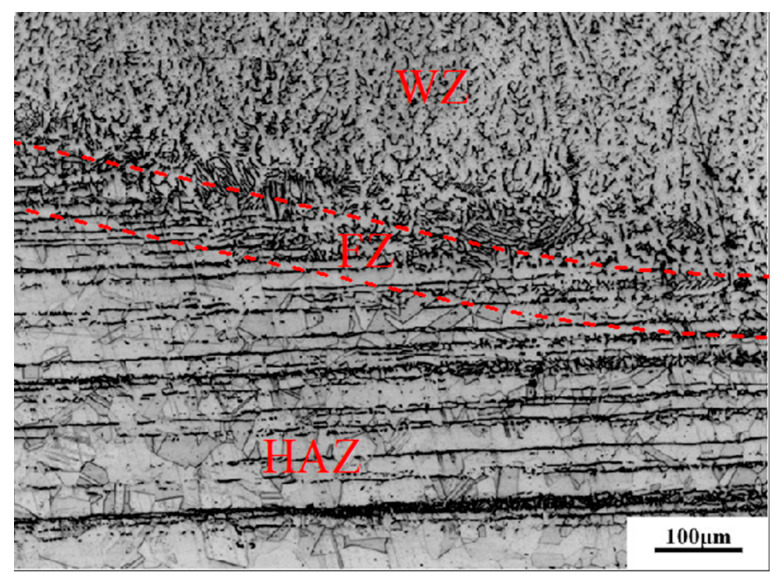
Metallographic structure of whole repair welding joint.

**Figure 7:**
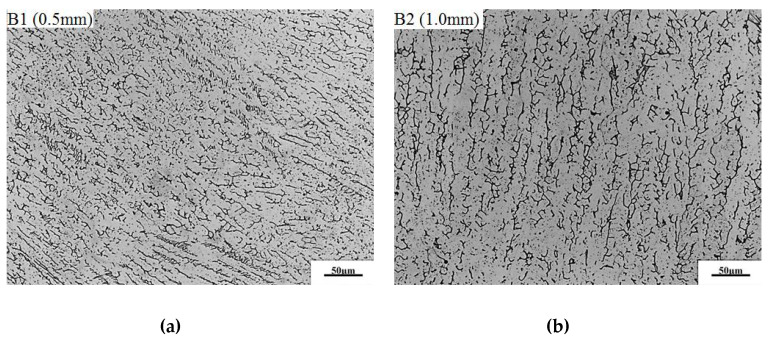

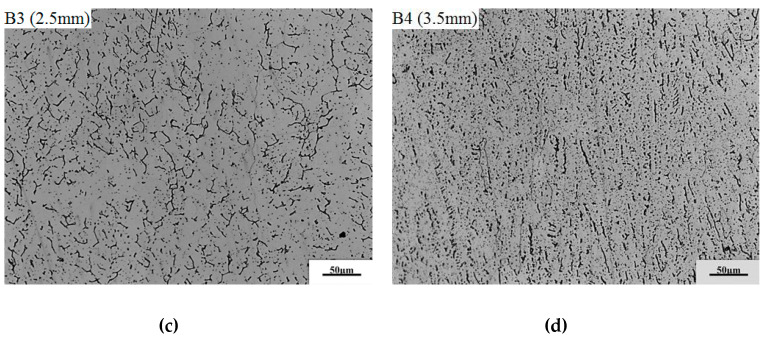
Effect of repair weld reinforcement height on the microstructure in the weld center: (**a**) 0.5 mm, (**b**) 1.0 mm, (**c**) 2.5 mm and (**d**) 3.5 mm.

Figure 6 shows the metallographic structure of the repair-welded joint containing the weld zone (WZ), fusion zone (FZ) and the heat affected zone (HAZ). It can be found that there is reticulated *δ* ferrite in such a weld, and a small amount of MC type carbide is distributed inside the austenitic grains. The fusion line is composed of plate-shaped delta ferrite distributed on the austenitic matrix. The microstructure in HAZ is composed of austenite grains and twins. The microstructures in three zones were compared respectively for different repair heights. Figure 7 shows the effect of repair weld reinforcement height on the microstructure in the weld center. There are many skeleton-like *δ* ferrite distributed on the austenite matrix for samples B1 and B2, while it is worm-like *δ* ferrite distributed on the austenite matrix for samples B3 and B4. Sample B3 and B4 contain two passes of welding, and the latter welding has the function of heat treatment for the previous weld. Because the former weld is heated again, the skeleton-like *δ* ferrite is dissolved and transformed, and breaks into interrupted worm-like *δ* ferrite. The contents of *δ* ferrite in the weld centers of samples B1, B2, B3 and B4 were 13.96%, 11.43%, 10.39% and 9.83%, respectively. The *δ* ferrite content in weld center decreases with the increases of the repair height.

**Figure 8:**
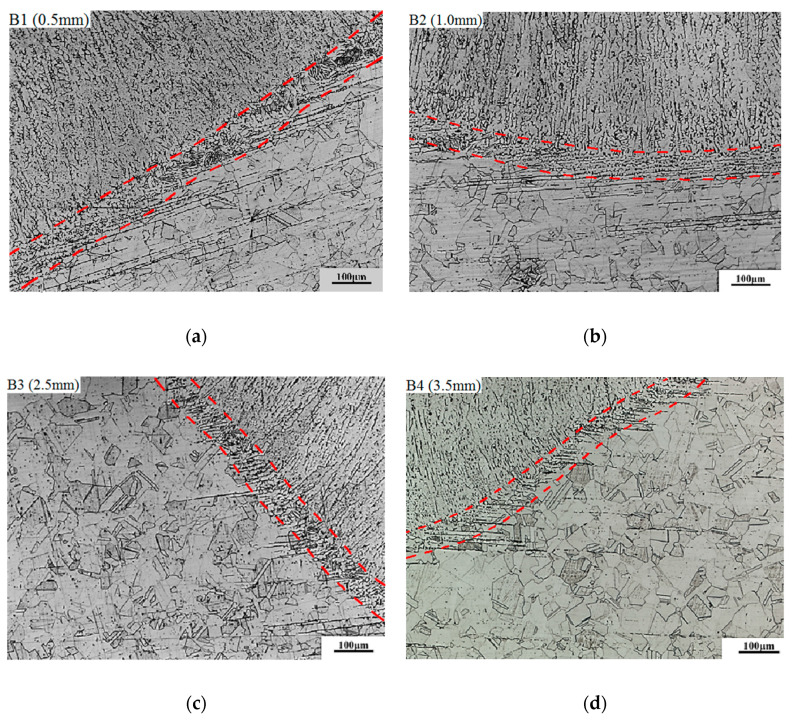
Effect of repair weld reinforcement height on the microstructure in the fusion line and the adjacent region: (**a**) 0.5 mm, (**b**) 1.0 mm, (**c**) 2.5 mm and (**d**) 3.5 mm.

Figure 8 shows the effect of repair weld reinforcement height on the microstructure in the fusion zone and adjacent region. The repair weld reinforcement height has little effect on the fusion line width and austenite grain size on the side of the base metal. With the increases of the repair weld reinforcement height, the banded *δ* ferrite content in the vicinity of the fusion zone is obviously reduced, and the ferrite shape is gradually changed from the skeleton to the worm shape. This is because when the repair weld reinforcement height increases, both the heat input and welding layer increase, resulting in the increases the heating time and number of the attachment structures of fusion line. Thus, the morphology and content of *δ* ferrite near the fusion line were changed.

**Figure 9:**
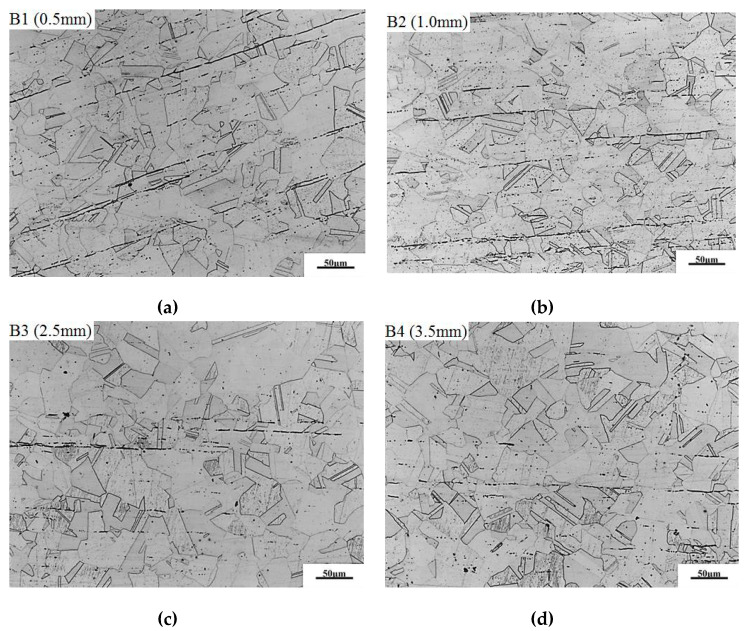
Effect of repair weld reinforcement height on the microstructure in the heat affected zone: (**a**) 0.5 mm, (**b**) 1.0 mm, (**c**) 2.5 mm and (**d**) 3.5 mm.

Figure 9 shows the effect of repair weld reinforcement height on the microstructure in HAZ. With the increases of repair weld reinforcement height, the austenite grain size has no changes, but the content of banded *δ* ferrite decreases gradually. The microstructure of HAZ is composed of austenite grain, twin and a few strips of *δ* ferrite. Generally speaking, the grain size of HAZ increases with increasing heat input and welding layer. However, due to the small thickness of the steel plate, the cooling rate of the repaired welding sample in air is faster, which leads to the austenite grain in HAZ not having enough time to grow.

**Figure 10:**
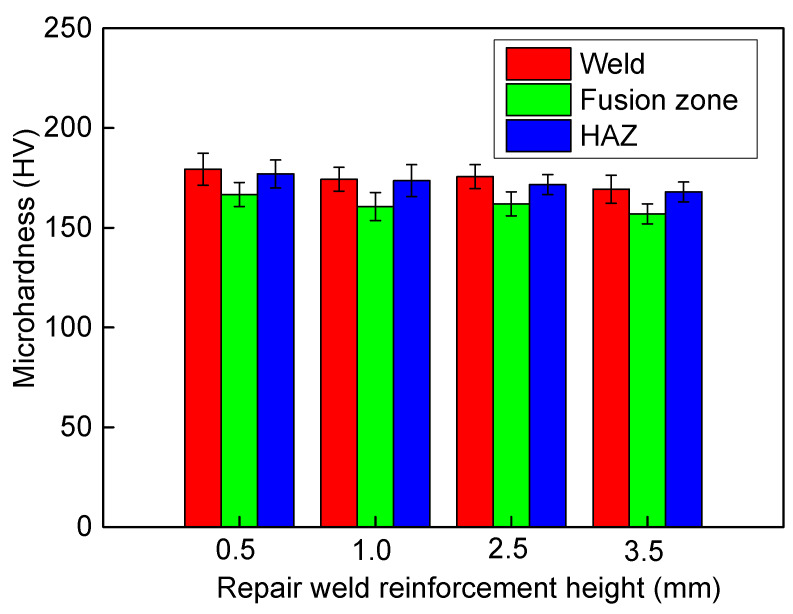
Effect of repair welding height on the microhardness.

Figure 10 shows the effect of repair welding height on the microhardness. The microhardness in the weld is the largest, followed by the heat affected zone, and the microhardness at the fusion line is the smallest. This is because the distribution of *δ* ferrite in weld zone is uniform, and the distribution of strip *δ* ferrite in heat affected zone is uneven. With the increase of welding reinforcement height, the Vickers hardness of weld and that of the heat affected zone decrease slightly. When the reinforcement height increases from 0.5 to 3.5mm, the microhardness in the weld and that in the HAZ decrease from 179 to 169 HV and 177 to 168 HV, respectively.

### 4.2. Analysis of Residual Stresses

**Figure 11:**
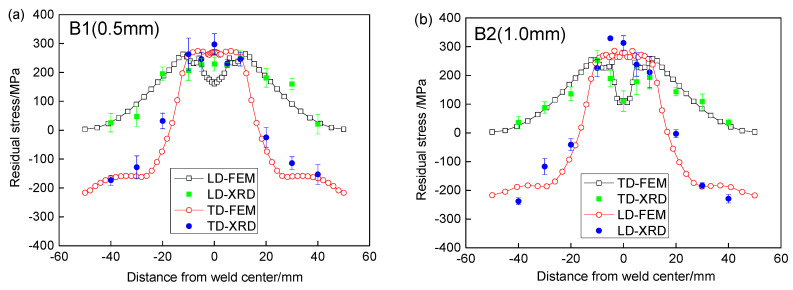

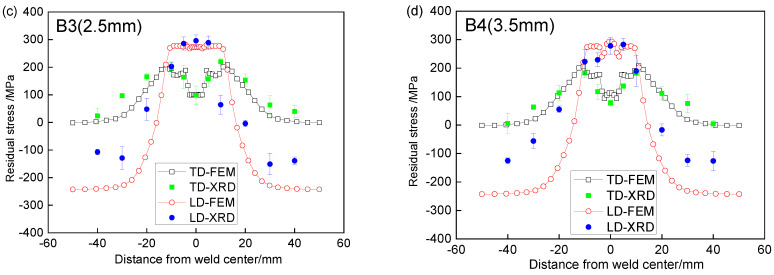
Comparison of residual stresses along P1 by simulation and experiment for different reinforcement heights: (**a**) 0.5 mm, (**b**) 1.0 mm, (**c**) 2.5 mm and (**d**) 3.5 mm.

Figure 11 the comparisons of residual stresses along P1 by finite element modeling (FEM) and X-ray diffraction (XRD). It is found that both the transverse and longitudinal residual stress by finite element analysis are in good agreement with those of XRD. The maximum error between simulations and experiments was 7%. It is shown that the numerical simulation method in this paper can predict the repaired residual stress of 304 stainless steel very well. There is a large tensile residual stress along P1 in the weld and HAZ for all repaired specimens, and the longitudinal residual stress is obviously larger than the transverse residual stress. The transverse residual stress is tensile stress and follows “M” distribution. The maximum transverse residual stress is located in HAZ and decreases gradually away from HAZ. The longitudinal residual stress is evenly distributed in the weld and heat affected zone, and gradually changes from tensile stress to compressive stress far from HAZ. With the increase of the repair welding reinforcement height, the longitudinal residual stress along P1 increases greatly, while the transverse residual stresses have no changes.

**Figure 12:**
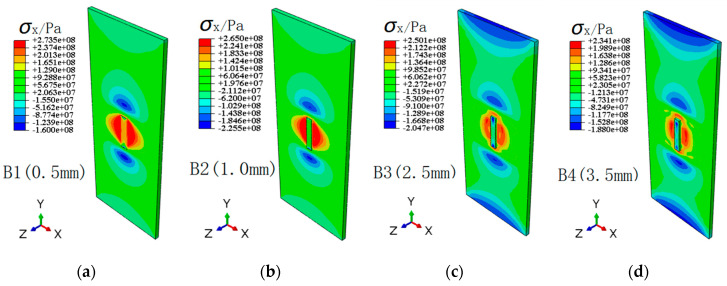
Contours of transverse residual stress for different repair weld reinforcement heights: (**a**) 0.5 mm, (**b**) 1.0 mm, (**c**) 2.5 mm and (**d**) 3.5 mm.

**Figure 13:**
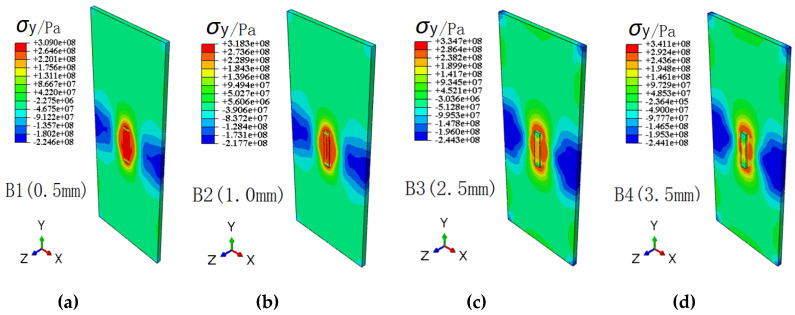
Contours of longitudinal residual stress for different repair weld reinforcement heights: (**a**) 0.5 mm, (**b**) 1.0 mm, (**c**) 2.5 mm and (**d**) 3.5 mm.

Figure 12 and Figure 13 show the contour distribution of transverse and longitudinal residual stresses for different repair weld reinforcement height, respectively. It is found that with the increases of repair weld reinforcement height, the maximum transverse residual stresses of repaired joint decrease, while the maximum longitudinal residual stresses increase. The maximum transverse residual stresses for repair weld reinforcement heights 0.5, 1.0, 2.5 and 3.5 mm are 273.5, 265.0, 250.1 and 234.1 MPa, respectively. However, the maximum longitudinal stress increases from 309 to 341 MPa when the repair weld reinforcement height increased from 0.5 to 3.5 mm. The transverse and longitudinal residual stresses in the weld decrease gradually, and the area of transverse and longitudinal tensile stress near the weld decreases gradually. However, the transverse compression stress zone distributed near the starting and ending point of the repair weld and the longitudinal compression stress zone on the left and right sides of the specimen increase with the increasing of repair welding height. Both the peak values of transverse and longitudinal tensile residual stress are located at the toe of the weld.

**Figure 14:**
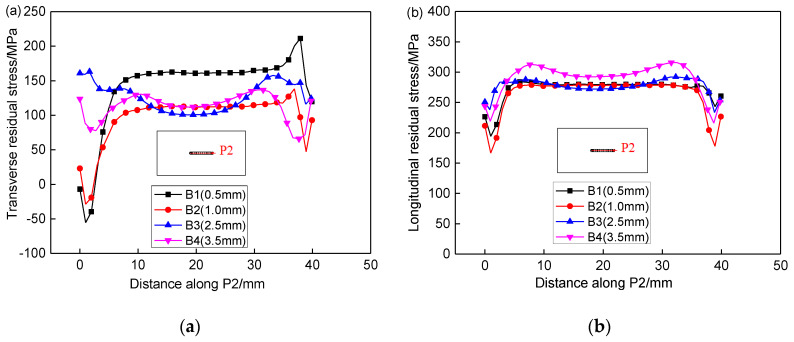
Effect of repair weld reinforcement height on the transverse (**a**) and longitudinal (**b**) residual stresses along P2.

Figure 14 shows the effect of repair weld reinforcement height on the transverse and longitudinal residual stresses along P2. When the repair weld reinforcement height increases from 0.5 to 1.0 mm, the transverse residual stress in the weld center decreases by about 40 MPa. When the repair weld reinforcement height is larger than 1 mm, the transverse residual stress in the weld center is basically maintained near 110 MPa. The transverse residual stress of sample B1 and B2 has a large gradient near the starting point of repair welding, which increases rapidly from −50 MPa at the starting point to about 150 MPa, and the transverse residual stress of samples B3 and B4 is distributed smoothly along P2. Except when the repair weld reinforcement height is 3.5 mm, there is little difference in the longitudinal residual stress for different repair weld reinforcement heights.

### 4.3. Analysis of SCC Sensitivity

**Figure 15:**
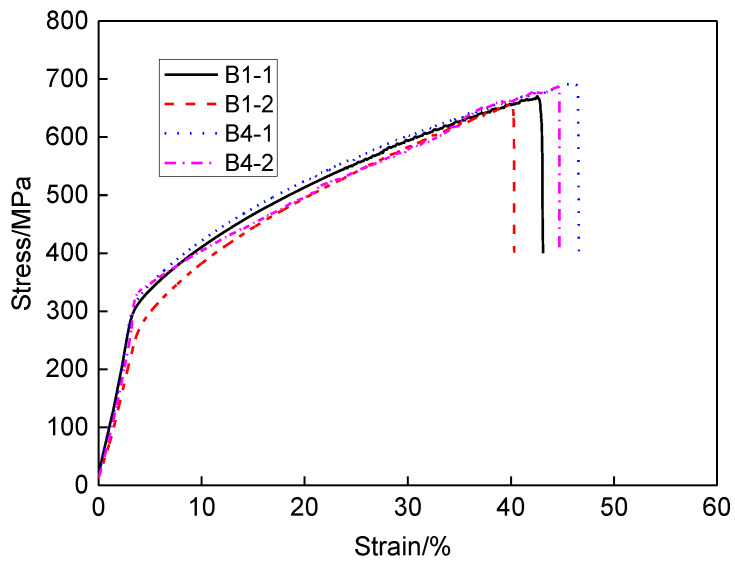
The SSRT curves for different solutions and dimensions.

By observing the fracture morphology of SSRT specimen, it can be found that the fracture position is located on the bottom edge of the original groove defect. The reason is that it is located between the weld and the base metal, and the mechanical properties in this position are weak. Figure 15 shows the SSRT curves for different solutions and dimensions. The symbosl B1-1 and B1-2 represent SSRT specimens with repair reinforcement height 0.5 mm placed in air and solution, respectively. The symbols B4-1 and B4-2 represent the SSRT specimens with repair reinforcement height 3.5 mm placed in air and solution, respectively. The tensile strength and elongation of the SSRT specimen with high repair weld reinforcement height are larger than those with low repair weld reinforcement height. The specimen B4 contains two welding passes, and the first pass will be reheated during next welding pass. Thus, the strength and elongation are increased. In the corrosion solution, the tensile strength and elongation of SSRT specimen are decreased compared to those in air. The SSRT results of for different specimens are all listed in Table 3. The SCC sensitivity indexes of repair reinforcement heights 0.5 and 3.5 mm are 6.34 and 3.91, respectively. That indicates that the higher the repair weld reinforcement height, the lower the SCC sensitivity of the repair sample (to a slight degree). Stress corrosion cracking is caused by corrosion medium, stress and other factors. The existence of residual stress can accelerate the initiation and propagation of SCC in the corrosive environment. As discussed in Section 3.2, the transverse residual stress for weld reinforcement height 3.5 mm is much lower than that for weld reinforcement height 0.5mm. Therefore, the SCC sensitivity with weld reinforcement height 3.5 mm becomes much weaker.

**Figure 16:**
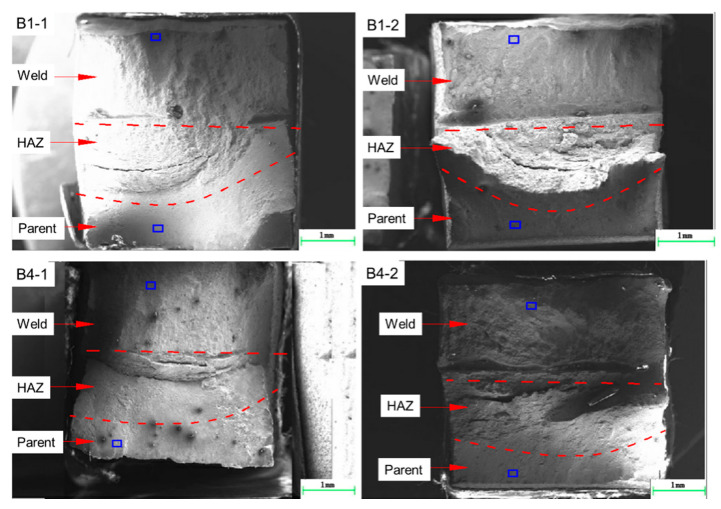
The macro-fracture surface morphologies of SSRT specimens.

Figure 16 shows the macro-fracture surface morphologies of SSRT specimens. It was found that the macroscopic fracture morphology in the weld, for all specimens, is relatively uniform and perpendicular to the direction of tensile stress. The fracture surface of in parent is also smooth, but it is at 45° to the direction of tensile stress. There are some macroscopic cracks in the heat affected zone (HAZ), showing brittle fracture characteristics. For the SSRT specimen in corrosion solution (B1-2 and B4-2), a long strip of grooves appear in the HAZ, which indicates that the plasticity near the fusion line is poor and sensitive to the corrosion medium.

**Figure 17:**
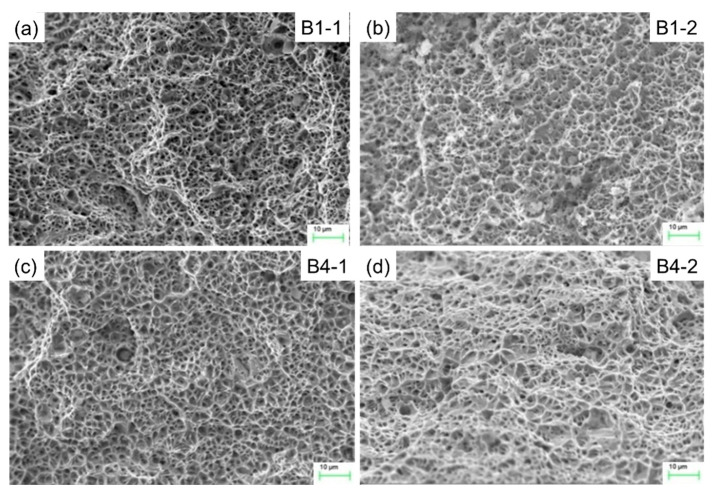
The micro-fracture surface morphologies in welds of SSRT specimens: (**a**) B1-1, (**b**) B1-2, (**c**) B4-1 and (**d**) B4-2.

**Figure 18:**
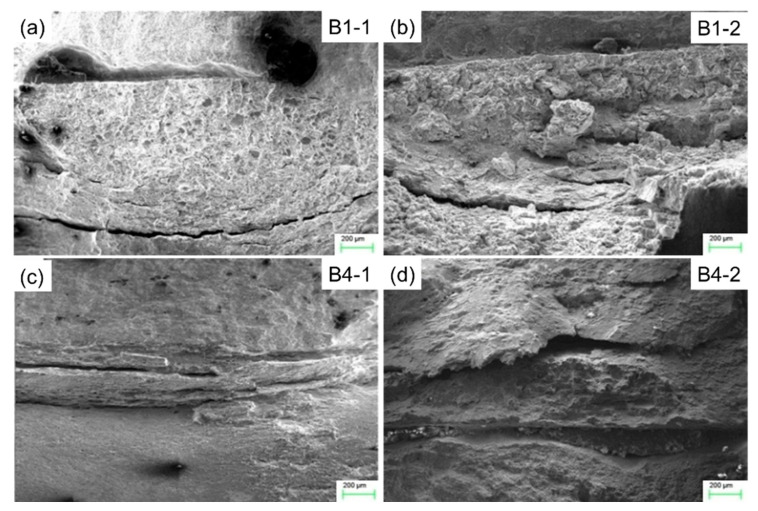
The micro-fracture surface morphologies in the heat affected zones (HAZs) of SSRT specimens: (**a**) B1-1, (**b**) B1-2, (**c**) B4-1 and (**d**) B4-2.

**Figure 19:**
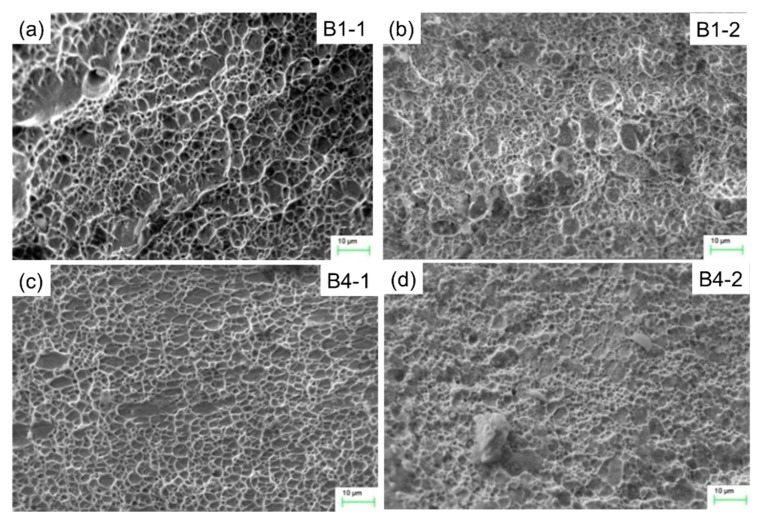
The micro-fracture surface morphologies in parent metal of the SSRT specimens: (**a**) B1-1, (**b**) B1-2, (**c**) B4-1 and (**d**) B4-2.

Figure 17, Figure 18 and Figure 19 show the micro-fracture surface morphologies of SSRT specimens in the weld, HAZ and parent metal, respectively. It was found that the microscopic fracture surfaces in welds of the four specimens show ductile fracture mode with typical dimple characteristics, as shown in Figure 17. By comparing the brightness in the fracture surface pictures, the dimples of specimens of higher reinforcement height are shallower than for the lower reinforcement height specimens.

Compared to the specimen that fractured in air, there are some torn edges in HAZs for the specimens fracturing in the corrosive solution, as shown in Figure 18b,d, which may be related to the SCC sensitivity of a HAZ to the corrosion medium. The fracture surface in the HAZ of a higher reinforcement height is more smooth compared to that of a shallow reinforcement height. It means that the reinforcement height has a certain influence on the fracture mode in HAZ.

As seen in Figure 19, the dimples in parent metal of specimens B1-2 and B4-2 are shallower than those of specimens B1-1 and B4-1, and the dimples in some areas are not obvious, indicating that the corrosion medium has some effects on the fracture mode of parent material. In addition, the dimples in parent metal of specimens B4-1 and B4-2 are shallower than those of specimens B1-1 and B1-2, respectively. That is to say, the SCC sensitivity of parent material is also influenced by the reinforcement height. Comparing Figure 17 and Figure 19, it can be seen that the dimples in the parent materials of all the samples are larger and shallower than those in the weld, indicating that the toughness of a parent metal is worse than that of the weld. In conclusion, the SCC sensitivity of the weld joint is mainly affected by the microstructure of the HAZ.

### 4.4. Discussion

Repair welding reinforcement height is an important parameter of weld pattern. In order to ensure the structure integrity of weld joint, the weld formation height is often higher than that of the parent material, so the weld reinforcement height is inevitably generated. The existence of the weld reinforcement height plays an important role in the heat preservation and the slow cooling of the whole welding seam, and is beneficial to refining crystal grains and reducing welding stress [40]. Based on above analysis, the *δ* ferrite content of higher reinforcement height is smaller than that of lower reinforcement height. The increased amount of the ferrite content will enhance the depth of corrosion pitting [7] and the risk of hydrogen embrittlement [41]. Thus, the microstructure and service performance of higher reinforcement height are superior to those of lower reinforcement height. On the other hand, the residual stresses are also decreased with the increases of repair reinforcement height. The smaller residual stress is beneficial to the inhibition of the failure of fatigue and SCC. Marcelino et al. [42] studied the effect of the number of repair welds on the fatigue strength of the welded joint of AIS4130 steel, and found that the fatigue strength of the local repair welding area is mainly affected by the metallographic structure and the microhardness. For the microhardness, the effect of repair reinforcement height is very small and can be ignored. Hence, a higher repair welding reinforcement height is recommended based on the considerations of microstructure and residual stress.

However, excess reinforcement height is not better. In general, the repair welding reinforcement height is generally increased with the increase of the thickness of the base metal, but not more than 3 mm [43]. At the same time, the weld reinforcement height is also the collection area of pores and other sundries, and results in local stress concentration [44]. Therefore, it is usually necessary to grind the reinforcement height of the weld to the same level as the base metal after repair welding. In this study, all the SSRT specimens are grinded to the same level. With the increases of reinforcement height, the tensile strength and elongation are all increased and the SCC sensitivity is decreased. Based on the consideration of corrosion sensitivity, the higher reinforcement height will be much better. However, for the higher reinforcement height, it will consume more material, repair time and become uneconomical. Therefore, the repair welding reinforcement height of 304 stainless steel should be controlled within 3.5 mm.

## 5. Conclusions

In this study, the effects of repair welding reinforcement height on microstructure, microhardness, residual stresses and SCC behavior of 304 stainless steel repaired joint were investigated by experiment and simulation. Additionally, an optimized repair welding reinforcement height was proposed. The following conclusions could be drawn:

- The penetration width and fusion width increase with the increases of heat input. With the increases of the repair weld reinforcement height, the *δ* ferrite content in weld and fusion zone is obviously reduced, and the ferrite shape is gradually changed from the skeleton to the worm shape.
- The microhardness from weakest to strongest is: weld, HAZ, and fusion zone. With the increases of repair welding reinforcement height, the microhardness of weld and heat affected zone decreases. The longitudinal residual stress in the weld is obviously larger than the transverse residual stress. There is an “M” distribution for the transverse residual stress.
- With the increases of repair welding reinforcement height, the transverse and longitudinal residual stresses in the weld decrease, and the area of the transverse and longitudinal tensile stress zone near the weld decreases. The effect of reinforcement height on longitudinal stress is larger than that of transverse stress.
- The tensile strength and elongation for higher repair weld reinforcement height are larger than those with lower repair weld reinforcement height. The higher the repair weld reinforcement height, the lower the SCC sensitivity of the repaired joint. It is ductile fracture mode with typical dimple characteristics in weld and parent material and brittle fracture mode in HAZ with macroscopic tear cracks.
- By combining the research results of Part I and Part II, the optimized repair welding parameters for 304 stainless steel were proposed: the repair welding heat input is recommended to be larger than 5KJ/cm and the reinforcement height is suggested to be controlled within 3.5 mm, based on the considerations of microstructure, hardness, residual stress and SCC sensitivity.

## Figures and Tables

**Table 1:** The chemical compositions of the parent metal and the filler wire (wt %).

Element	C	Si	Mn	P	S	Cr	Mo	Ni	Cu
Parent	0.048	0.419	1.228	0.031	0.0018	18.08	0.011	8.113	0.0096
Weld	0.053	0.5	1.78	0.028	0.003	18.96	0.26	10.21	0.41

**Table 2:** Welding parameters of repaired specimens with different repair heights.

Specimen No.	Repair Length	Repair Height	Welding Layer	Voltage	Current	Welding Speed
	*L*/mm	*k*/mm	*n*	*U*/V	*I*/A	*v*/(mm/s)
B1	40	0.5	1	20	110	3
B2	40	1.0	1	20	110	2.5
B3	40	2.5	2	20	90	3
B4	40	3.5	2	20	90	2.5

**Table 3:** The SSRT results of for different solutions and dimensions.

Specimen No.	Tensile Strength *R*_m_/MPa	Fracture Time *t*/h	Elongation *δ*/%	SCC Sensitivity Index *I_δ_*/%
B1-1	670.28	170.98	43.06	6.34
B1-2	656.76	161.20	40.33	
B4-1	691.80	184.78	46.51	3.91
B4-2	687.80	176.82	44.69	
